# Death from Thirty Grains of Dover’s Powder

**Published:** 1882-07

**Authors:** S. Bailey


### ARTICLE IV.

### DEATH FROM THIRTY GRAINS OF DOVER'S POWDER.

The following case is reported by Dr. S. Bailey, of Mt. Ayr, Iowa, and occurred in the practice of a neighboring physician.

The patient was Mrs. G. W.; forty-two years old; white; born in Eastern Illinois; has lived here four years. Was a housewife; married; has had five or six children, the youngest three months old. Was a woman of strong constitution; general health good; no hereditary predisposition; never had any serious illness or injury. Habits of life regular; hygienic surroundings good. Since the birth of her youngest child, had been quite unwell; has never ceased "to flow," and has had at times very severe pains; to use the family's own words, "her pains were just the same as if she was in labor." These would occur frequently. At times she would be morose and gloomy; ordinarily she was gay and happy.

On March 31, patient complained of having a cold and cough, and could not rest. Her physician was called, and found patient about as described, and prescribed five powders, each containing five grains powdered muriate of ammonia, and ten grains Dover's powder, two of which she took Friday night. She slept most of the night, and the next morning still felt quite drowsy, but was roused by her nurse to eat some breakfast, sitting up in bed; went to sleep again, and slept till ten o'clock A. M., when her nurse roused her, and gave her another powder. The patient went to sleep soon; in fact, right away, and slept till twelve M., when her nurse again roused her. This required some effort, as she was quite sleepy and dull, but was roused sufficiently to allow her babe to nurse. She again went to sleep, and remained so till between three and four o'clock P. M., when the family tried to rouse her and could not succeed, when another physician was called, who pronounced her narcotized from opium. The usual remedies were resorted to, such as active movement, rubbing, and strong coffee, without producing any effect whatever. At nine P. M. I was called; at that time her pulse was 118 and strong; respiration about twenty-five per minute, and stertorous; the face was livid, lips blue, and extremities cold; skin moist and cool; pupils contracted, and the eyes flexed or turned to the right the greater part of the time; also turned up. I applied a battery, and kept it up till 3:30 next morning, the 2d. During the interval from nine till twelve her condition remained unchanged, except the respiration became more hurried, increasing from twenty to fifty or sixty per minute, till she died, which took place at five o'clock A. M. the 2d of April. At 2:30 I administered a hypodermic injection of three drops of acetous tr. nux vomica. Spasmodic action of the muscles of arms and legs, the peculiar effect of nux vomica, was produced. This did not change the circulation, and did not seem to produce any general effect except to increase the already hurried respirations.

